# Evaluation of aerial spraying application of multi-rotor unmanned aerial vehicle for *Areca catechu* protection

**DOI:** 10.3389/fpls.2023.1093912

**Published:** 2023-02-28

**Authors:** Juan Wang, Chao Ma, Pengchao Chen, Weixiang Yao, Yingbin Yan, Tiwei Zeng, Shengde Chen, Yubin Lan

**Affiliations:** ^1^ College of Mechanical and Electrical Engineering, Hainan University, Haikou, China; ^2^ National Center for International Collaboration Research on Precision Agricultural Aviation Pesticides Spraying Technology, College of Electronic Engineering and Artificial Intelligence, South China Agricultural University, Guangzhou, China; ^3^ Guangdong Laboratory for Lingnan Modern Agriculture, Guangzhou, China; ^4^ College of Information and Electrical Engineering, Shenyang Agricultural University, Shenyang, China; ^5^ Liaoning Engineering Research Center for Information Technology in Agriculture, Shenyang, China; ^6^ Patent Examination Cooperation Guangdong Center of The Patent Office, China National Intellectual Property Administration (CNIPA), Guangzhou, China; ^7^ College of Information and Communication Engineering, Hainan University, Haikou, China

**Keywords:** droplet deposition, multi-rotor UAV, LAI, *Areca catechu* protection, aerial spray, spray drift

## Abstract

Multi-rotor unmanned aerial vehicle (UAV) is a new chemical application tool for tall stalk tropical crop *Areca catechu*, which could improve deposit performance, reduce operator healthy risk, and increase spraying efficiency. In this work, a spraying experiment was carried out in two *A. catechu* fields with two leaf area index (LAI) values, and different operational parameters were set. Spray deposit quality, spray drift, and ground loss were studied and evaluated. The results showed that the larger the LAI of *A. catechu*, the lesser the coverage of the chemical deposition. The maximum coverage could reach 4.28% and the minimum 0.33%. At a flight speed of 1.5 m/s, sprayed droplets had the best penetration and worst ground loss. The overall deposition effect was poor when the flight altitudes were greater than 11.09 m and the flight speed was over 2.5 m/s. Comparing flight speed of 2.5 to 1.5 m/s, the overall distance of 90% of the total drift increased to double under the same operating parameters. This study presents reference data for UAV chemical application in *A. catechu* protection.

## 1 Introduction

*Areca catechu* is native to the tropical Pacific, Southeast Asia, South Asia, and East Africa and belongs to the palm family of perennial evergreen trees. It is grown mainly in India, China, Indonesia, and Africa (Ansari et al., 2021; Salehi et al., 2020). According to relevant statistics, in 2019, the global *A. catechu* planting area was 1,240,253 ha, with a total production of 1,722,273 tons. In 2019, the area under *A. catechu* cultivation in China Hainan Island was 115,171 ha, with a total production of 287,043 tons; it is the province’s primary economic source for more than 2.3 million farmers. According to statistics, *A. catechu* has more than 40 diseases and 100 insect pests, and among the diseases, *A. catechu* yellowing disease is the most serious (Cao et al., 2021; Wang H. et al., 2020). It is a systemic disease of the bast, affecting leaves and flowers. Bacterial leaf spots, fruit rot, and anthracnose mainly affect the fruit, heart leaves, foliage, and other parts of the areca fruit growing on the tree trunk. Reviewing references and field surveys shows that the general *A. catechu* disease sites occur on the leaf surface and the heart of the leaf and trunk parts. The height of *A. catechu* can reach 10–20 m after entering maturity, and the height of 7–8-year-old trees cultivated with new varieties in recent years can reach 8–9 m. *A. catechu* mainly grows in hilly areas, making the application more complicated. The canopy of branches and leaves overlap each other, and nuts are in the trunk position; these growth forms and pest and disease characteristics make it necessary for pesticide spraying. *A. catechu* can be manually protected and applied during the seedling period, but the difficulty of manual application increases after the tree height exceeds 2 m. Most farmers adopt rough management after the height of *A. catechu* is greater than 3 m.

The application of unmanned aerial vehicles (UAVs) provides new viable tools for crop protection (Li et al., 2021). As a new technology, UAVs offer an alternative to ground machines and manual knapsacks, especially for complex terrain and tall trees (Giles, 2016). In the Asia Pacific, UAV application technology can help alleviate the growing labor shortage caused by aging farm populations (Matthews, 2019; Wang G. et al., 2020). Japan began using Yamaha RMAX radio-controlled helicopters for rice pest and disease control in the 1990s (Sheets, 2018). In the United States, UAV spraying is slowly integrating into commercial agriculture, especially in specialized application scenarios such as the treatment of invasive weeds, pest control for vineyard spraying on steep terrain, and fungicide application in the air (Lan et al., 2010; Giles and Billing, 2014; Biglia et al., 2022). In California, RMAX is approved for vineyard-controlling diseases on grape foliage (Giles and Billing, 2015; Giles, 2016). In China, labor shortages in the countryside have accelerated the need to use UAVs for crop spraying (He, 2018). Regarding spraying effect and efficiency, UAV sprayers have absolute advantages in wheat, rice, and other crops while reducing the exposure risk of sprayers (Faiçal et al., 2014; Faiçal et al., 2017; Mogili and Deepak, 2018). Commercial applications of low-altitude, low-volume UAV sprayers are multiplying in East Asian countries such as China, Japan, and South Korea. Especially in China, UAVs are used on low crops such as rice, wheat, corn, and cotton and even on some economic crops such as apples, tea, pineapples, lychees, and sweet potatoes (Meng et al., 2019; Vougioukas and Rutledge, 2019). Most electric multi-rotor UAVs have limited payloads and more flexible and autonomous flight control. These UAVs are rapidly commercialized in related fields and are receiving increasing attention worldwide. For example, the multi-rotor UAV P20 (Guangzhou XAG Technology Co., Ltd.) has an intelligent control system and multi-directional radar mode and uses precision spraying, uniform seeding, and intelligent mapping.

Incorporating UAVs as a new form of plant protection into modern commercial crop protection systems requires extensive research. Evaluation indicators include the effectiveness of pest and disease control and the degree of environmental pollution. The environmental meteorology, wind speed, and temperature indicated on the pesticide instructions are generally suitable for shaped ground or aerial application equipment. Since UAVs are emerging products, these instructions do not specifically mention UAVs. Many factors may influence the coverage, droplet size, and drift potential of UAV spraying, such as the selection of appropriate meteorological conditions, flight altitude, flight speed, nozzle types, and droplets size (Huang et al., 2019; Teske et al., 2019; Wang J. et al., 2019). Spray quality requires to achieve better droplet coverage on crops. Spray drift is defined as droplets evaporating off the target crop, stagnating, or depositing at a distant ground level during or shortly after pesticide application (Chen et al., 2022). The droplet sizes of UAVs are approximately 270–350 μm, the droplet size of ground-based machinery is approximately 300–1,000 μm (Yallappa et al., 2017; Ling et al., 2018), and smaller droplets (less than 200 μm in diameter) have a higher risk of drift (Chen et al., 2022). However, finer droplet sizes can achieve better spray coverage, which contradicts reducing drift. In addition to particle size, flight altitude and speed, ambient wind speed, temperature and humidity, and rotor wind fields influence spray quality and drift (Wang et al., 2022; Zhan et al., 2022). Application environments such as orchards, tall trees, and vast canopies are challenging for any application method, especially when farmers need higher spray penetration and effectiveness for the target area. Therefore, it is essential to pay attention to the risk of drift while discussing the spray quality of crops.

Areca trees in Hainan Island are primarily grown in hilly areas. Farmers usually use knapsack sprayers in the seedling stage, with low efficiency, high intensity, and potential pesticide poisoning risk. Due to the planting density and terrain limitation, it is difficult for ground application machines to operate. There have been little research data on the mechanized application of *A. catechu* globally. Further research is needed to determine whether UAVs can achieve the expected deposition and penetration to achieve the desired level under a high leaf area index (LAI) due to the small UAV loads and low spraying volumes. The specific objectives of this study were to a) acquire more data to assess the effectiveness of UAVs as spraying devices for tall crops, b) evaluate the leaf area index on the distribution of droplet deposition inside the canopy for different aerial application parameters on *A. catechu*, and c) evaluate the environmental impact of UAV application in tall crops, including ground loss and drift.

## 2 Materials and methods

### 2.1 UAV and spray system

**Figure 1:**
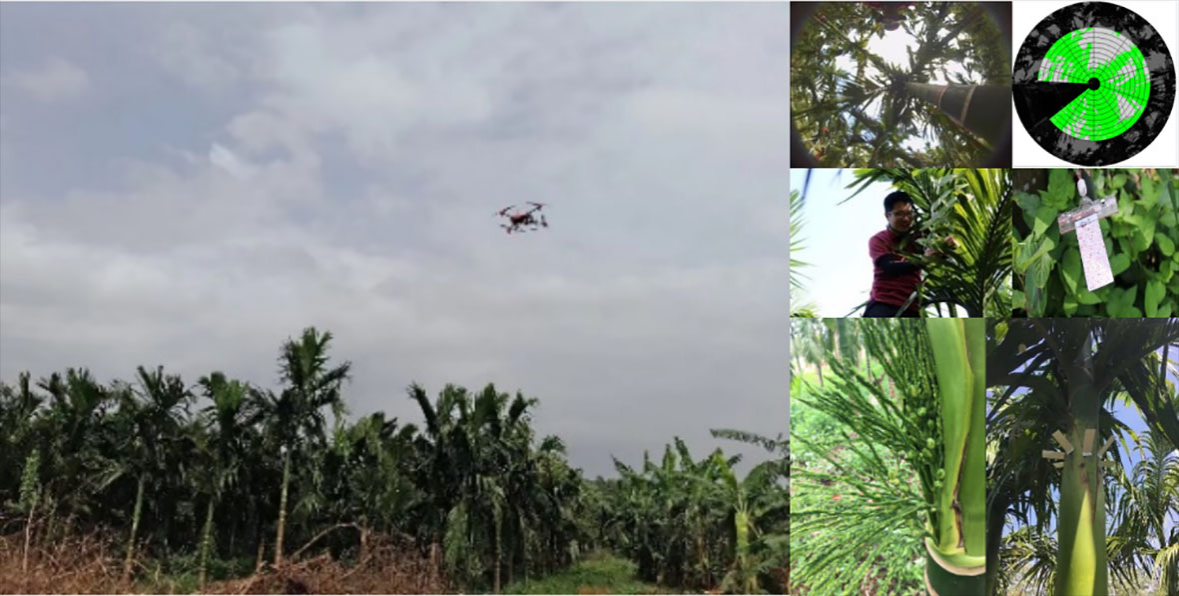
The layout of the treatment area by UAV spraying areca test. UAV, unmanned aerial vehicle.

This experiment used a P20 multi-rotor UAV (Guangzhou XAG Science and Technology Co., Ltd., Guangzhou, China) with a SUPERX2 RTK flight control system. Furthermore, a more accurate GNSS RTK positioning module spraying system makes UAV spraying more intelligent, accurate, and efficient **(** **Figure 1** **)**. It can map farmland boundaries and intelligently plan high-precision routes. With modular design, it can be interfused between a fixed base station, mobile base station, and handheld mapper, with simple operation and quick surveying and mapping of farmland by one person **(** **Table 1** **)**. The accuracy of UAV route planning can reach a centimeter level, realizing high-precision flight routes. The NK-5500 Kestrel weather meter (NK, USA) performs environmental and meteorological monitoring, collecting data every 5 s, including ambient temperature and humidity, wind speed, and wind direction. CI-110 Plant Canopy Image Analyzer (US CID Company, Camas, WA, USA) obtained the LAI of *A. catechu*, which was taken vertically upward from the bottom of the plant according to the instructions of the instrument. The instrument connects to a handheld tablet computer, which can display test images in real-time. The BeiDou system (an aerial BeiDou positioning UB351 system developed by the South China Agricultural University with the RTK differential positioning function) collects UAV flight trajectory, and the horizontal accuracy reaches (10 + 5 × D × 10^−7^) mm. The elevation accuracy reaches (20 + 1 × D × 10^−6^) mm, where “D” represents the distance value measured by the system. The data collection interval was 0.1 s. The BeiDou system can record the flight trajectory and precise operation time of the UAV (Yao et al., 2019).

**Table 1:** Parameters for UAV application.

Main parameter	UAV
Aircraft type	Jifei P20
Dimensions (m)	1.18 × 1.18 × 0.41
Nozzle type	Centrifugal nozzle
Spray width (m)	1.5–3
Nozzle numbers	4
Flow rate (ml/min)	200–800
Spray height (m)	1–10
Driving speed (m/s)	1–8
Tank capacity (L)	6, 8, 10
Spraying pattern	Low volume and high concentration
Power system	B12710 smart battery

UAV, unmanned aerial vehicle.

### 2.2 Testing layout

The test site is located at the *A. catechu* demonstration base (National Center for International Collaboration Research on Precision Agricultural Aviation Pesticides Spraying Technology) in Chengmai County, Hainan Province (19°57′57″N, 110°08′58″E). The tree was 5–6 years old before flowering and fruiting. The tree height ranged from 4.7 to 6.3 m, the planting density was 1,800 plants/ha, the leaf area index ranged from 0.81 to 1.91, and the row spacing was 2.0 m × 2.5 m.

### 2.3 Sampling arrangement

**Figure 2:**
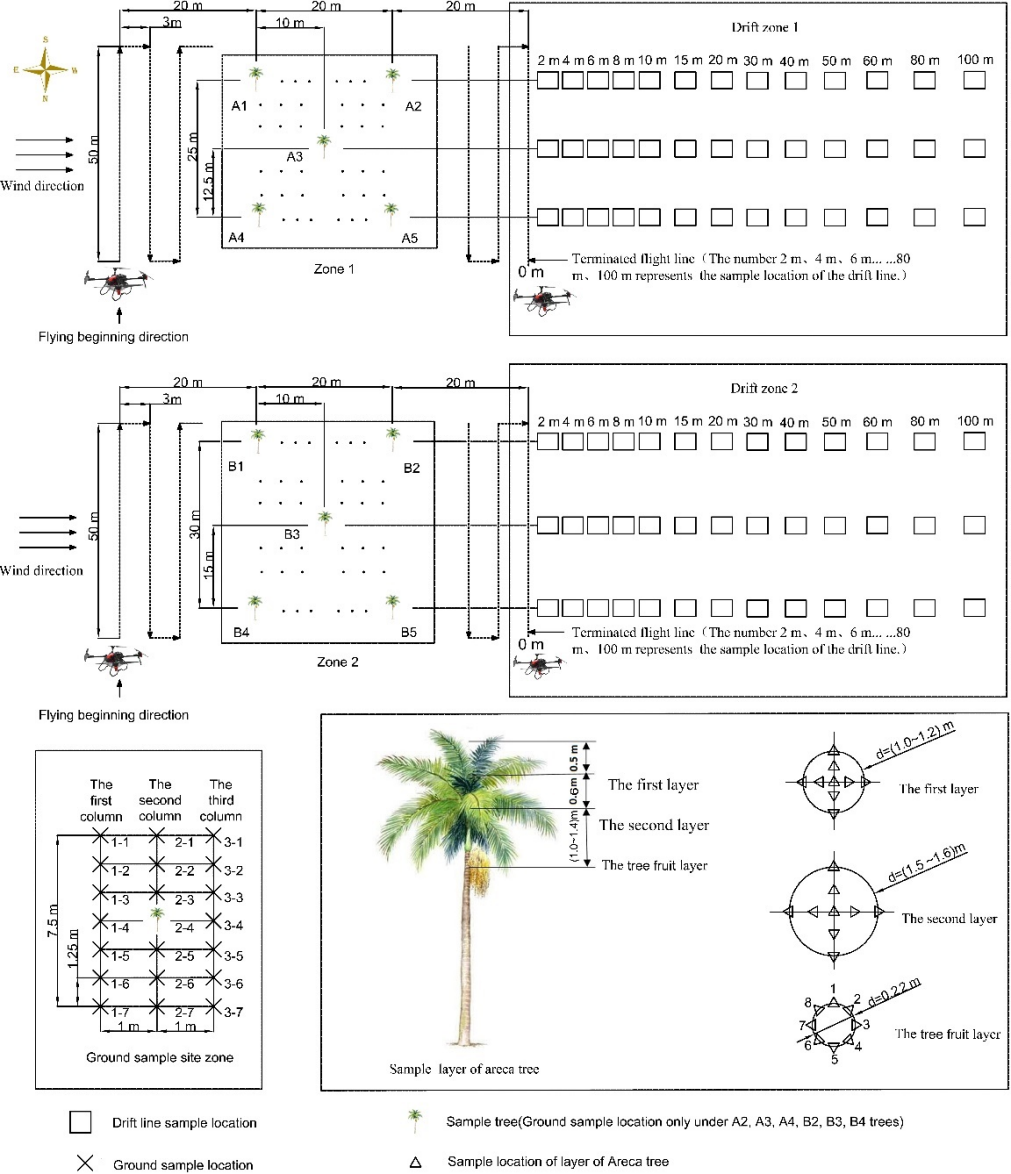
The layout of the test plan for spraying *Areca catechu* by UAV. UAV, unmanned aerial vehicle.

The planting form, canopy structure, fruit location, and plant growth shape of the *A. catechu* differed from those of low crops such as rice and wheat. The spraying volume should be based on the amount of pesticide required by crop biomass per unit volume rather than the land area size per unit. Leaf area index and vegetation biomass are characteristic parameters of crop growth status information, which can use as an essential basis for controlling the amount of pesticide spraying. At the same spraying rate, crops with different leaf area indices had different droplet deposition results. The pre-experimental data showed that the deposition had a significant coefficient of variation (Yao et al., 2019 **)**. In order to test the data more accurately and objectively, the experiment divides into two zones for 5–6-year-old trees. As shown in **Figure 2**, five plants with similar leaf area indexes were selected for the test according to X type. The average leaf area index of plants was 1.01 and the average tree height was 4.61 m in zone 1, and they were 1.65 and 4.96 m, respectively, in zone 2. The sampling was divided into three sections: canopy, ground, and drift (Wang J. et al., 2020) (the reference model is another DJI multi-rotor model, and the two test programs are the same; for the specific model, please refer to the reference).

#### 2.3.1 *Areca catechu* sampling arrangement

The top of the *A. catechu* canopy has a small transverse area, and the angle between the leaves and the trunk is small, which is more prone to pests and diseases. The middle part has the largest transverse area, the angle between the leaves and the trunk increases, and the branches and leaves are dense, which was the critical control area. The trunk layer was the location of nut fruiting, which was the critical area for pest and disease prevention when flowering and fruiting. Due to the above reasons, we sampled three layers in the canopy, the upper layer was approximately 0.5 m from the top canopy, and the angle between the leaves and the trunk was approximately 30°–50°. The sampling cards were arranged in a “+” shape; the center was arranged as one sampling point; in each of the four directions, two sampling points, east, south, west, and north, were arranged at equal distances; and a total of nine sampling points were arranged in the layer. Compared with the traditional five sampling points for pests and diseases, there were four more sampling points, and the diameter of this layer was approximately 1.0–1.2 m. The middle layer was approximately 0.6 m from the upper layer, with a diameter of 1.5–1.6 m, and the arrangement was the same as that of the upper layer. The third layer was on the trunk, with a diameter of approximately 0.22 m and 1.0–1.4 m from the middle layer; around the circumference arranged, eight sampling points were at equal intervals. The sampling points were marked in advance with numbered white filter paper for accurate sample recovery and placement. Each flight recovered a total of 260 sampling cards.

#### 2.3.2 Ground loss sampling arrangement

The ground loss sampling of the two LAIs of *A. catechu* was arranged in six rectangular areas (2 × 7.5 m), with every three areas replicated as a group. Three collection lines are arranged in the upwind, canopy interception, and downwind areas. Each area contained one sampled tree, i.e., A2, A3, A4, B2, B3, and B4. Each collection line arranged seven sampling points, with the arrangement method and spacing shown in **Figure 2**. A sharpened polyvinyl chloride (PVC) pipe was inserted into the ground, a universal clip was placed approximately 30 cm above the ground, and sampling cards were placed on the universal clip. For each flight, a total of 126 sampling cards were recovered.

#### 2.3.3 Sedimentation drift sampling arrangement

The drift collection area was divided into two areas with three repetitions. The first area was arranged on the extension line of the sampling *A. catechu* with an average LAI of 1.01 and the second area with an average LAI of 1.65. The spray drift start position was 2 m to the right of the ending position of the spraying area, and the ending line was set at 0 positions; a total of 13 sampling points were set up sequentially at 2, 4, 6, 8, 10, 15, 20, 30, 40, 50, 60, 80, and 100 m. The sampling points were arranged in the same way as the ground sampling arrangement. Each sampling point was marked in advance with filter paper for accurate placement and recovery of droplet collection cards. Each flight collected a total of 78 cards.

### 2.4 Operation parameters and spraying liquid configuration

Allura Red tracer at 5‰ was selected instead of pesticide spraying. The test was sorted six times,

and the test was repeated in the exact location of the same plant. The droplet sampling card was made of copper plate card with a size of 75 × 25 mm. The test variables were flight altitude and speed of the UAV and LAI of *A. catechu*, and the spraying volume was 22.5 L/ha.

The UAV operation mode was consistent with the actual operation in the field. The UAV took off from the top of the first row of the areca tree in the field, with a spray width of 3 m, a flight length of 50 m, and a total width of flight area of 60 m. The UAV took off and landed 20 m away from the sampling area. The test was divided into 12 treatments. **Table 2** shows the operating and environmental parameters of the test processing. The operating altitude and speed of the UAV in the table were all obtained from the BeiDou RTK positioning system.

**Table 2:** Operating parameters and environmental information.

No. of test	Mean flight speed (m/s)	Mean flight height (m)	Mean temperature (°C)	Mean humidity (%)	Mean wind speed and direction (m/s)
Treatment 1	1.50	8.84	23.50	63.70	1.26/Southeast
Treatment 2	1.50	8.84	23.50	63.70	1.28/Southeast
Treatment 3	1.50	10.31	22.30	68.20	1.55/Southeast
Treatment 4	1.50	10.31	23.30	68.20	1.54/Southeast
Treatment 5	1.50	11.09	22.20	67.70	1.20/Southeast
Treatment 6	1.50	11.09	22.20	67.70	1.25/Southeast
Treatment 7	2.50	8.84	25.00	60.30	1.87/Southeast
Treatment 8	2.50	8.84	25.00	60.30	1.78/Southeast
Treatment 9	2.50	10.31	23.90	62. 00	1.47/Southeast
Treatment 10	2. 50	10.31	23.90	62.00	1.45/Southeast
Treatment 11	2.50	11.09	23.20	65.50	1.87/Southeast
Treatment 12	2.50	11.09	23.20	65.50	1.78/Southeast

### 2.5 Data processing method

After each flight execution, when the sampling cards were completely dry, the testers wore disposable gloves, collected the pre-numbered sampling cards in the designated envelopes, placed them in sealed bags, and store them in ice boxes. After the test, the scanned images were analyzed with the processing software Deposit Scan to obtain the droplet deposition, droplet size, number of droplets, and coverage.

#### 2.5.1 Coefficient of variation

The coefficient of variation describes the degree of variation of the same data group. This test describes the variation range of droplet deposition, coverage, and droplet size of each sample in the same group. The larger the coefficient of variation, the worse the uniformity. The uniformity of distribution mentioned below is calculated based on this, and the coefficient of variation (CV) calculation formula is as follows:


$$\begin{array}{c} CV=\frac{S}{\bar{X}}\times 100\%\\ S=\sqrt{\sum_{\text{i}=1}^{\text{n}}{\left(X_{i}-\bar{X}\right)}^{2}/(n-1)} \end{array}$$

where *S* is the standard deviation; *X*_*i*_ is the deposition (μl/cm^2^), coverage (%), and droplet volume median diameter (μm); $\bar{X}$ is the deposition of each sample (μl/cm^2^), coverage (%), and droplet volume median diameter (μm); and *n* is the number of sampling points in each group.

#### 2.5.2 Deposition level

Deposition level represents the percentage *k* (%) of droplet deposition in the amount of application.


. $$k=\frac{\beta_{dep}}{\beta_{v}}\times 10,000$$

where *β*_*dep*_  is the droplet deposition (μl/cm^2^) and  *β*_*v*_  is the spray volume per hectare (L/hm^2^).

#### 2.5.3 Drift rate and percentage

The droplet drift deposition level calculation is the same as in Section 2.5.2. According to the ISO22866 standard, the total measured value of spray drift *β*_*T* _(100%) is calculated by the following formula:


. $$\beta_{T}=\int_{2}^{100}\text{k}\left(\text{x}\right)\text{dx}$$

A 90% drift distance is defined as the distance that reaches 90% of the total spray drift test amount in meters.

## 3 Results and analysis

### 3.1 Spray quality

#### 3.1.1 Canopy deposition distribution

**Figure 3:**
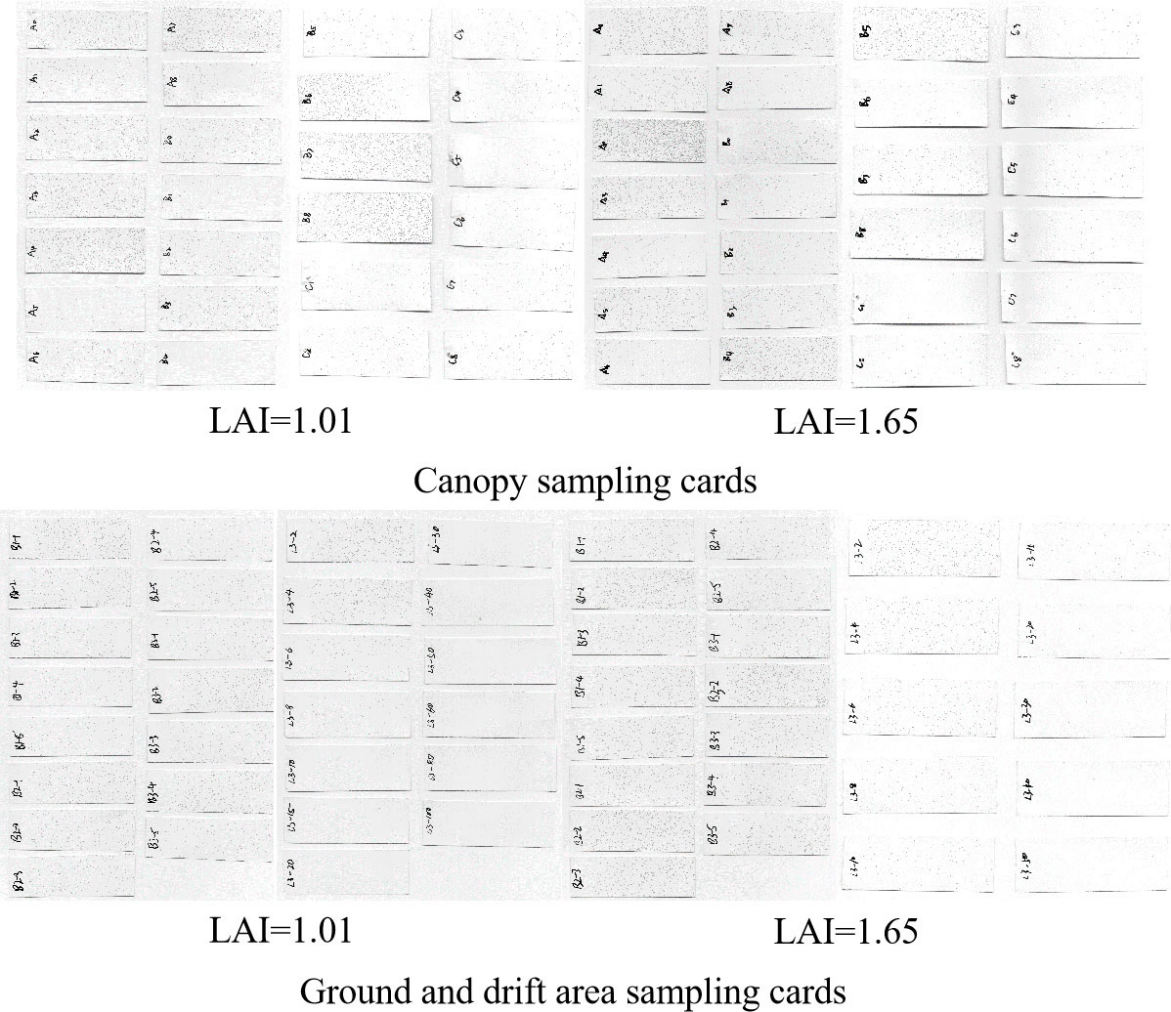
Partial sampling cards of *Areca catechu* canopy, ground, and drift areas.

During the experiment, the sampling cards were placed according to the angle of leaf growth. The distribution of droplets on the sampling card can approximate the deposition of droplets on the leaf surface, and the difference of each processing data can approximate the influence of the experimental variables on deposition. The scanned image of the sampling card is shown in **Figure 3**. Areca plantation was sprayed by the UAV with six operational parameters and recorded using a copper plate card, as shown in the figure with sampled images of canopy, ground, and drift areas. The data results showed a trend consistent with the visual observations.

#### 3.1.2 Spray coverage

**Figure 4:**
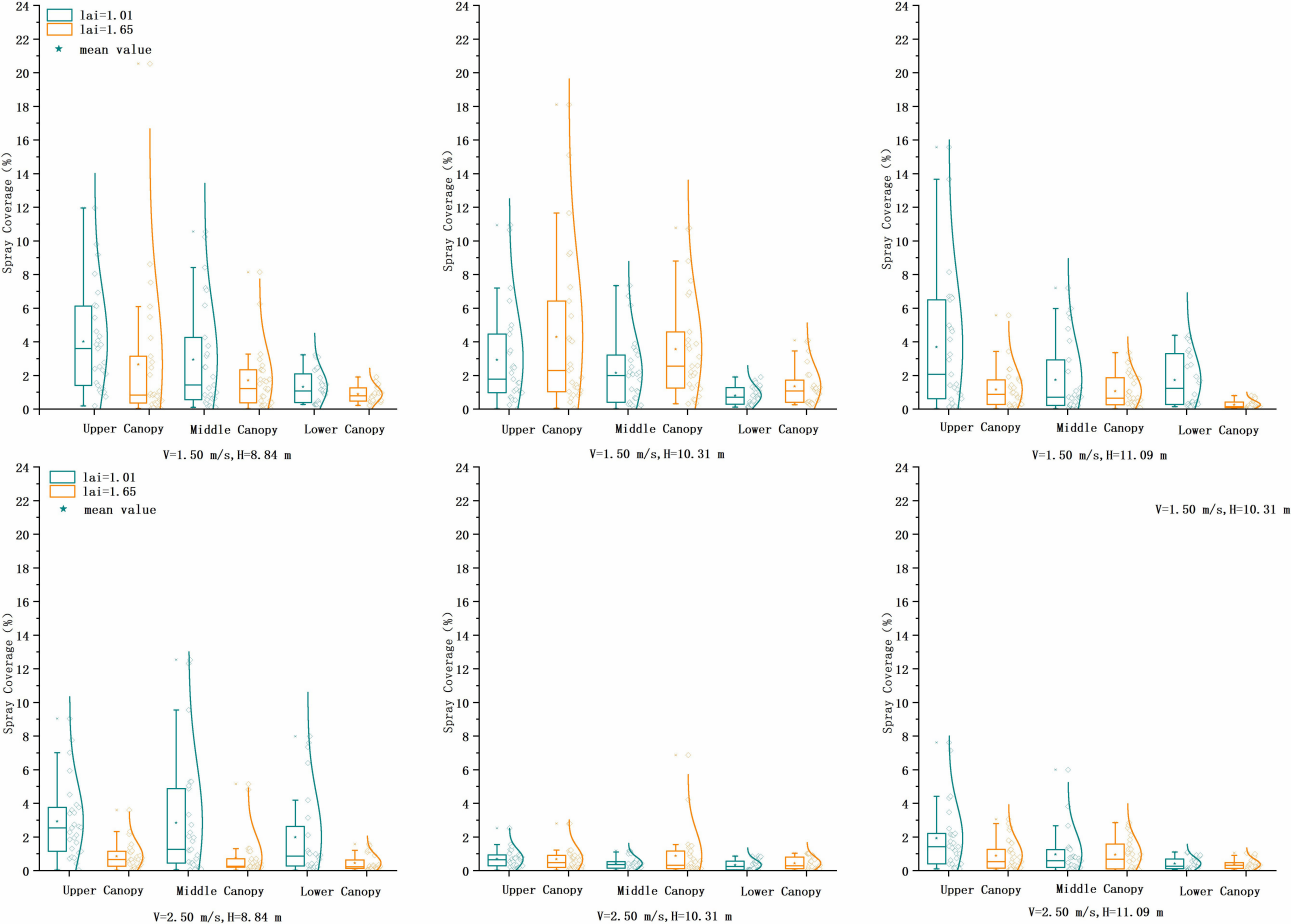
Comparison of spray area coverage of copper plate cards at two flight heights and three flight speeds at three heights in the canopy of two LAI plants. LAI, leaf area index.

The UAV sprayed on two LAI areca plants at two flight speeds and three flight altitudes. **Figure 4** shows the spray coverage recorded on the sampled copper plate cards. The data analysis showed no statistically significant difference in the coverage for each treatment (*p* > 0.05), which was different from the visual effect. The results showed that treatment 4 (LAI = 1.65, v = 1.5 m/s, h = 10.31 m) had the highest total spray coverage, and treatment 9 (LAI = 1.01, v = 2.5 m/s, h = 10.31 m) had the lowest. The total canopy coverage was significantly lower for both different LAI trees at the same flight altitude with a speed of v = 2.50 m/s compared to v = 1.50 m/s. At LAI = 1.01, the coverage decreases were 7% (H = 8.84 m), 75% (H = 10.31 m), and 54% (H = 11.09 m). At LAI = 1.65, the coverage decreases were 61% (H = 8.84 m), 78% (H = 10.31 m), and 11% (H = 11.09 m).

At LAI = 1.01 and v = 1.5 m/s, the flight altitude was 10.31 m compared to 8.84 and 11.09 m, and the total coverage decreased by 29% and 18%, and at v = 2.5 m/s, the total coverage decreased by 82% and 57%. At LAI = 1.65 and v = 1.5 m/s, the flight altitude was 10.31 m compared to 8.84 and 11.09 m, and the total coverage increased by 75% and 18%. At v = 2.5 m/s, the uniformity of coverage distribution improved but decreased more. The variability of droplet penetration inside the canopy was expressed in coverage CV values, ranging from 0.20 to 0.69. Only at the operating height of 11.09 m was there a significant difference in the penetration of spray coverage of the two LAI in the interior of the canopy, with v = 1.5 m/s, and CV values were 47% (LAI = 1.01) and 59% (LAI = 1.65), and at v = 2.5 m/s, the CV values were 69% (LAI = 1.01) and 43% (LAI = 1.65).

The larger the LAI value, the denser the branches and leaves, and the lower the spray coverage. The upper canopy had a maximum coverage of 4.28% and minimum coverage of 0.68%. The middle canopy had a maximum coverage of 3.56% and minimum coverage of 0.78%. The lower canopy had a maximum coverage of 1.98% and minimum coverage of 0.33%. Analysis of the full canopy spray coverage showed that when LAI = 1.01, v = 1.5 m/s performed better than v = 2.5 m/s, with an overall coverage increase of approximately 70%. When v = 2.5 m/s, the coverage of height 8.84 m was 5.3 and 2.3 times higher than that of the other two heights. When LAI = 1.65, v = 1.5 m/s performed better than v = 2.5 m/s, with an overall coverage increase of approximately 172%.

The UAVs have two modes of spraying fruit trees, hovering and full coverage. Due to the small diameter of the UAV rotor relative to the tree canopy, the more common full-coverage spraying method was used in this experiment. The UAV uses a counter-rotating rotor running at different speeds to produce a wind field that can directly affect the trajectory of the droplet. The downward wind field can help the droplets penetrate the canopy better and improve penetration. The precise relationship between wind field and droplet motion theory is still under study. How to select appropriate operating parameters and the correct use of downward wind will have an important impact on droplet penetration. The penetration effect of the UAV in the canopy of areca trees in this test is satisfactory.

The distribution of canopy branches and leaves was complex. It was difficult to infer the precise relationship between spray coverage and flight height due to the complex growth pattern of *A. catechu* trees and the test height of only three groups. The coverage information on the copper plate card can qualitatively describe the distribution of spray deposition within the tree canopy. However, the sample collection area was limited relative to the leaf area and needed to be supplemented with ground and drift area sampling data. The spray coverage data showed that the overall performance at UAV flight altitude was below 10.31 m, and the speed was 1.5 m/s, which can provide data references for UAV application to tall trees in tropical areas.

Uniform coverage of small droplets is considered to increase the effectiveness of pest control. The range of canopy coverage CV values indicates the degree of heterogeneity, with CV values ranging from a minimum of 54% to a maximum of 163%. The lower canopy was more uniformly distributed, related to the size and number of droplets that can move to the trunk layer. Relatively speaking, when pest control is focused on the upper canopy, larger droplet sizes can be selected, with coarse droplets more inclined to deposit upon the first impact with the foliage and smaller droplets more likely to penetrate the lower canopy and trunk. It is essential to recognize that more droplets, even at lower concentrations, are preferable to fewer droplets at much higher concentrations. Spray coverage is vital to ensure effective pest and disease control, and the high temperatures and high humidity conditions of Hainan Island, China, may present evaporation and drift risks. Different droplet particle sizes can be selected as needed for practical applications.

#### 3.1.3 Droplet size

There was no significant difference in droplet size (*p* > 0.05) between the upper canopy and the lower canopy except for treatment 3 (*p*< 0.05). The upper layer data showed that only treatment 1 significantly differed from treatment 6 and treatment 10 (*p*< 0.05). There was no significant difference between the middle and lower layers for the other treatments (*p* > 0.05). The droplet sizes of all treatments were divided into 630 groups for significance analysis, and only 47 groups were found significantly different (*p*< 0.05). Significant differences occurred when the operating parameters, canopy position, and LAI were changed simultaneously, mainly in the upper canopy (treatment 1) and lower canopy (treatments 3, 5, 6, 9, 11, and 12). The data analysis in **Table 3** showed that when LAI = 1.01, the droplet size in the lower canopy was different from the other sampling layers, with the smallest particle size (190 μm), which may be related to the density of branches and leaves in the canopy of *A. catechu*.

**Table 3:** Droplet size characteristics and distribution uniformity.

Flight test no.	Flight parameters	Sample location	DV50(µm)				
			Mean(µm)	SD	CV (%)	Min(µm)	Max(µm)
	LAI = 1.01 V = 1.50 m/s H = 8.84 m	Upper layer	255.52	18.90	7.40	218.33	279.33
Test 1		Middle layer	245.48	29.75	12.12	204.00	291.67
		Lower layer	212.63	6.47	3.04	201.67	220.33
	LAI = 1.65 V = 1.50 m/s H = 8.84 m	Upper layer	233.15	23.68	10.16	201.00	283.00
Test 2		Middle layer	248.11	84.00	33.86	167.33	463.00
		Lower layer	207.00	12.80	6.18	183.67	225.00
	LAI = 1.01 V = 1.50 m/s H = 10.31 m	Upper layer	242.19	17.82	7.36	219.33	268.33
Test 3		Middle layer	229.96	16.27	7.07	199.00	249.67
		Lower layer	187.33	8.70	4.64	178.00	199.33
	LAI = 1.65 V = 1.50 m/s H = 10.31 m	Upper layer	240.41	24.22	10.08	192.00	274.00
Test 4		Middle layer	232.07	19.69	8.49	190.33	253.33
		Lower layer	206.79	9.82	4.75	187.00	221.67
	LAI = 1.01 V = 1.50 m/s H = 11.09 m	Upper layer	228.15	30.03	13.16	164.33	260.67
Test 5		Middle layer	208.07	33.87	16.28	162.00	249.33
		Lower layer	194.92	19.22	9.86	163.33	216.33
	LAI = 1.65 V = 1.50 m/s H = 11.09 m	Upper layer	205.89	28.96	14.06	141.00	246.33
Test 6		Middle layer	221.85	36.60	16.50	181.33	309.00
		Lower layer	183.17	9.13	4.98	167.00	192.67
	LAI = 1.01 V = 2.50 m/s H = 8.84 m	Upper layer	241.56	15.62	6.47	206.33	256.33
Test 7		Middle layer	250.22	13.90	5.55	230.00	268.67
		Lower layer	228.21	37.90	16.61	167.00	258.67
	LAI = 1.65 V = 2.50 m/s H = 8.84 m	Upper layer	217.00	15.40	7.10	193.00	237.67
Test 8		Middle layer	214.59	22.69	10.57	170.33	242.00
		Lower layer	218.08	18.04	8.27	186.33	245.67
	LAI = 1.01 V = 2.50 m/s H = 10.31 m	Upper layer	208.20	8.27	3.97	190.00	219.33
Test 9		Middle layer	203.85	15.42	7.57	173.67	219.00
		Lower layer	184.06	23.41	12.72	145.33	216.33
	LAI = 1.65 V = 2.50 m/s H = 10.31 m	Upper layer	201.70	25.74	12.76	162.67	241.33
Test 10		Middle layer	204.67	21.43	10.47	173.00	236.00
		Lower layer	200.38	22.01	10.98	167.00	238.67
	LAI = 1.01 V = 2.50 m/s H = 11.09 m	Upper layer	239.74	15.91	6.64	206.00	262.00
Test 11		Middle layer	221.41	18.66	8.43	200.33	247.67
		Lower layer	190.25	15.43	8.11	162.67	213.33
	LAI = 1.65 V = 2.50 m/s H = 11.09 m	Upper layer	227.37	18.64	8.20	201.00	255.67
Test 12		Middle layer	203.22	31.36	15.43	141.67	241.00
		Lower layer	185.67	27.58	14.86	155.00	244.33

LAI, leaf area index.

Rotor downwash airflow and windward and crosswind airflow had coupling effects on each other. They changed the canopy pore distribution state and affected the droplet movement law, forcing the droplet movement track to change, the droplet to be captured by the leaves, or the pores to continue moving forward. The larger the droplet size, the greater the inertia force, and the less affected the droplets are by an air drag force. The droplets cannot easily penetrate the inner canopy leaf pore space, while the smaller the particle size, the stronger the ability to change the track transport. The data showed that the droplet size of the upper canopy was the largest and the lower canopy was the smallest under the same treatment, which again indicated that the smaller droplet size had a more vital variable orbit ability and could easily penetrate the canopy and reach the bottom.

The droplet size was analyzed for all treatments, with mean values of 235.89 μm for the upper canopy, 226.50 μm for the middle canopy, and 199.57 μm for the lower canopy at LAI = 1.01. At LAI = 1.65, the mean value of the upper canopy was 219.45 μm, that of the middle canopy was 220.75 μm, and that of the lower canopy was 200.18 μm. The mean size of ground loss droplets was 232.92 μm.

#### 3.1.4 Number of droplets

**Figure 5:**
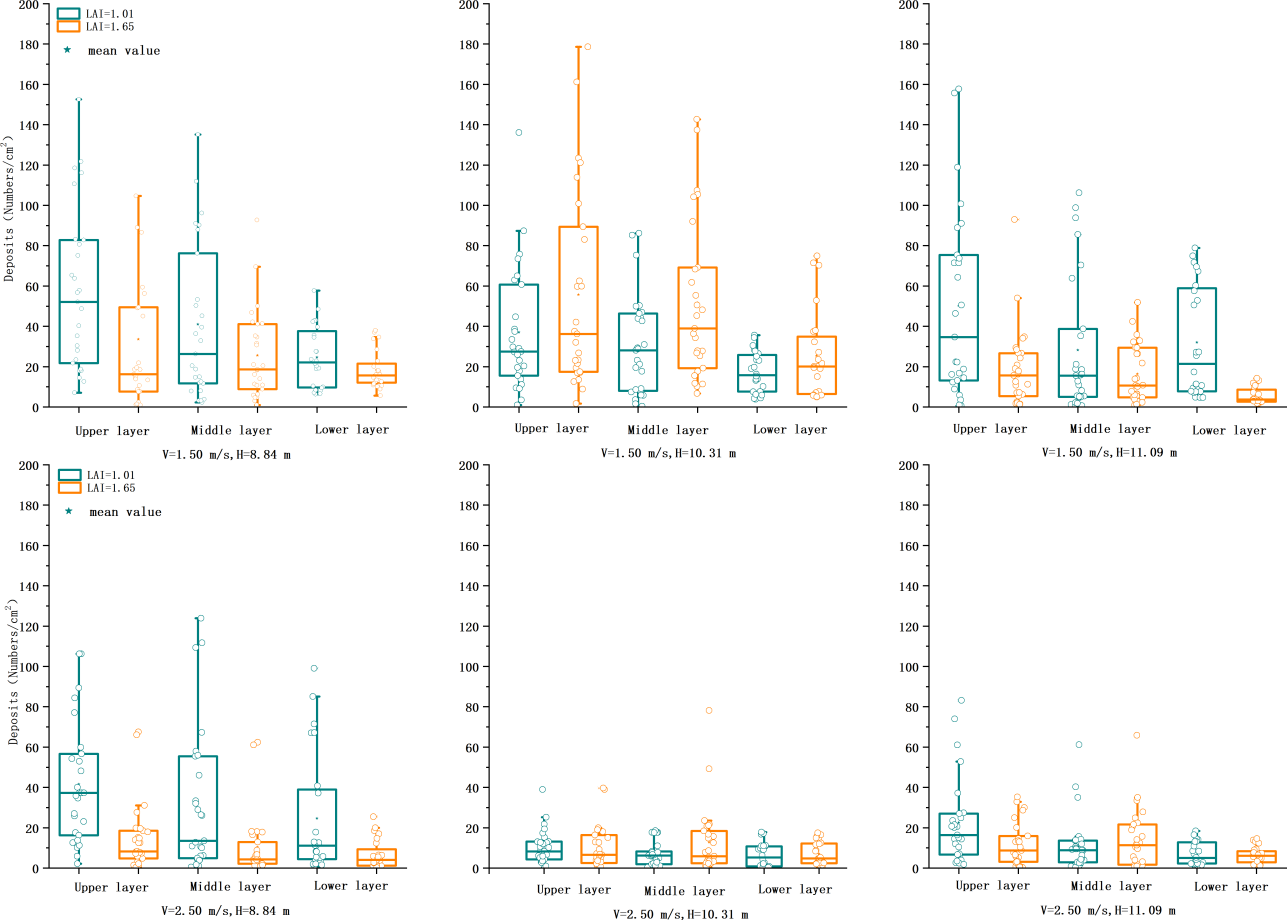
Comparison of deposition of copper plate cards at two flight heights and three flight speeds at three heights in the canopy of two LAI plants. LAI, leaf area index.

**Figure 5** shows the number of canopy droplets, and the analysis showed that treatment 1 had the highest number of droplets at LAI = 1.01 with an average of 43.81 droplets/cm^2^, and treatment 9 had the lowest of 8.25 droplets/cm^2^. Treatment 4 had the highest number of droplets at LAI = 1.65 with an average of 44.62 droplets/cm^2^, and treatment 12 had the lowest number at 10.06 droplets/cm^2^. At v = 2.5 m/s, the number of droplets was reduced by about half compared with that at v = 1.5 m/s. The performance was poorer at a flight speed of 2.5 m/s, and the number of droplets was higher at v = 1.5 m/s and below an altitude of 10.31 m. The higher the flight altitude, the more the droplet deposition shape expands, and the droplet distribution is more uniform in a particular altitude range. The faster the flight speed, the shorter the rotor’s downwash wind field is in affecting the time on the droplet. A higher height of the wake vortex will increase its duration, resulting in the droplet motion track being more randomly and quickly affected by the surrounding environment.

#### 3.1.5 Droplet deposition

**Figure 6:**
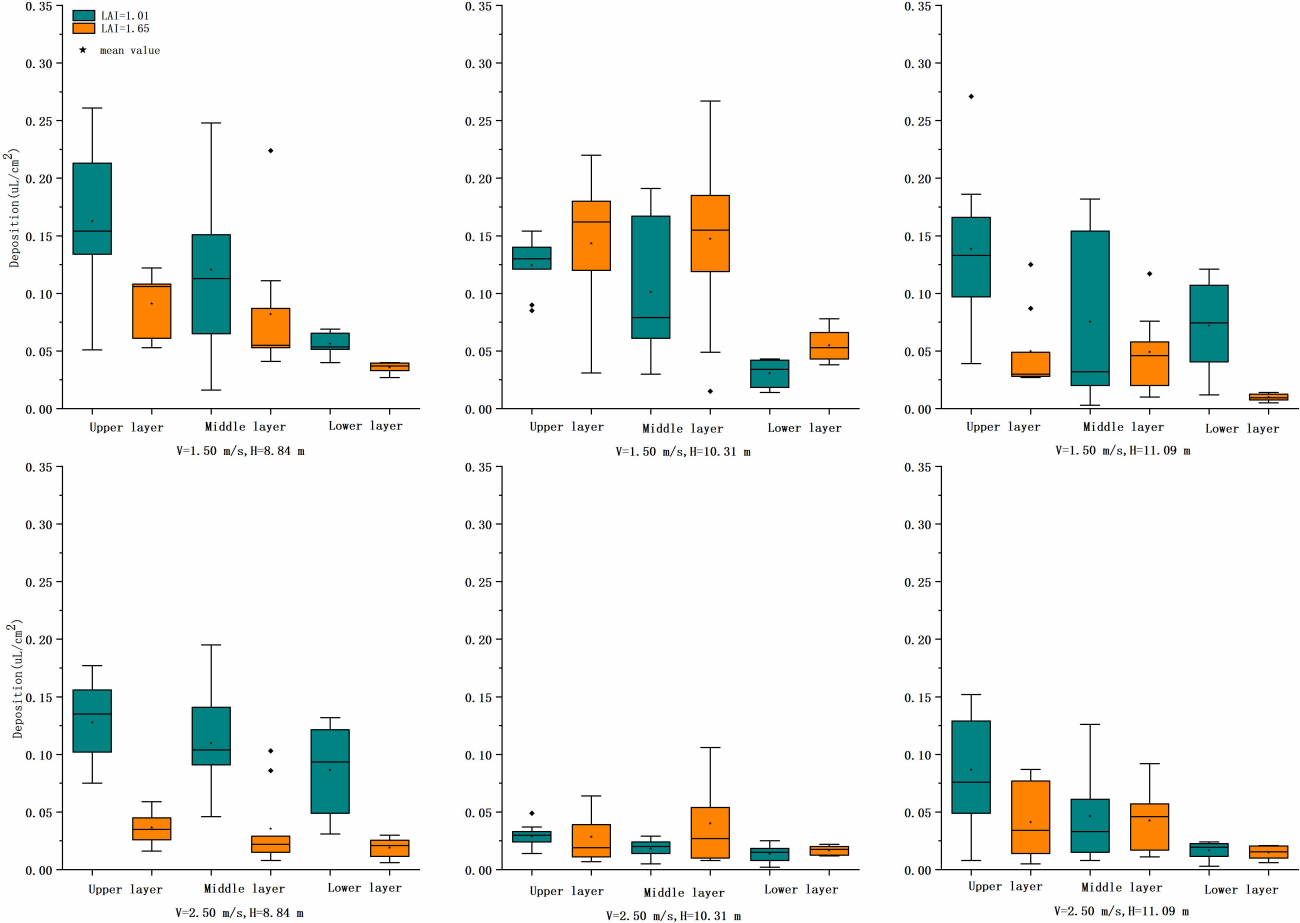
Comparison of the number of droplets at two flight heights and three flight speeds at three heights in the canopy of two LAI plants. LAI, leaf area index.

*A. catechu* canopy size and branch density significantly impact droplet movement, and the instability of droplets reaching the target increases as tree height, canopy depth, and density increase. The release height of aerial application droplets of tall trees was higher, and the time of droplet movement in the air grew. The interaction between the rotor downwash wind field and the canopy crop pore structure affected the droplets below the UAV. The droplets caused by the rotor tip vortex were mainly distributed in the outer circumference with smaller particle size, reduced kinetic energy, and increased stagnation time. The influence of the rotor downwash wind field had been gradually weakened when the droplets reached the interior of the canopy, which was consistent with the results of Hong et al. (2018). The amount of droplet deposition at different canopy locations is shown in **Figure 6**.

##### 3.1.5.1 Upper canopy

At LAI = 1.01 and v = 1.5 m/s, the deposition was higher at all three flight heights; at up to 0.16 μl/cm^2^ and v = 2.5 m/s, the deposition reached 0.13 μl/cm^2^ at 8.84-m height and decreased rapidly above 10.31-m height. By Tukey’s test, treatment 1 was significantly different from treatment 11 (*p*< 0.05), and treatment 9 showed highly significant differences compared with treatments 1, 3, 5, and 7 (*p*< 0.01). The higher speed and altitude of UAV operation parameters significantly affected the deposition of droplets in the upper canopy. Overall, treatment 1 had the highest mean value of 0.16 μl/cm^2^, and treatment 9 had the lowest value of 0.03 μl/cm^2^.

At LAI = 1.65, the mean deposition was 0.09 and 0.14 μl/cm^2^ at treatment 2 and treatment 4, respectively, and the mean deposition of the rest of the treatments was close to and lower, with treatment 10 having the lowest at 0.03 μl/cm^2^. By Tukey’s test, the deposition showed a significant difference (*p*< 0.05) between the altitude of 10.31 m and the others for v = 1.5 m/s and had no significant difference (*p* > 0.05) for v = 2.5 m/s. In terms of application parameters, both LAIs were more suitable for flight altitudes below 10.31 m, with v = 1.5 m/s. If the flight altitude or operating altitude increased, both decreased the deposition significantly.

##### 3.1.5.2 Middle canopy

At LAI = 1.01 and v = 1.5 m/s, the deposition amount was higher at 10.31-m height; at up to 0.12 μl/cm^2^ and v = 2.5 m/s, the deposition amount reached 0.11 μl/cm^2^ only at 8.84-m height and decreased rapidly above 10.31-m height, down to 0.02 μl/cm^2^. Tukey’s test showed that treatment 9 significantly differed from treatments 1, 3, and 7 (*p*< 0.05); i.e., there was a significant effect on the deposition in the middle layer at this speed and height. Overall, treatment 1 had the highest mean deposition value of 0.12 μl/cm^2^, and treatment 9 had the lowest value of 0.02 μl/cm^2^.

At LAI = 1.65, the mean deposition was 0.08 and 0.15 μl/cm^2^ only in treatment 1 and treatment 2, respectively, while the mean deposition of the other treatments was close to and lower than 0.04 μl/cm^2^. By Tukey’s test, there was a significant difference (*p*< 0.05) between the deposition at 10.31- and 11.09-m height at v = 1.5 m/s, and there was no significant difference (*p* > 0.05) between the treatments at v = 2.5 m/s. In terms of application parameters, it was found that both LAIs were more suitable for operation at heights below 10.31 m and speed at 1.5 m/s again.

##### 3.1.5.3 Lower canopy

At LAI = 1.01 and v = 1.5 m/s, the highest mean deposition was 0.07 μl/cm^2^ (H = 11.09 m), and the lowest was 0.03 μl/cm^2^ (H = 8.84 m). By Tukey’s test, treatment 3 and treatment 5 showed a significant difference (*p*< 0.05); that is, there was a significant effect on the deposition of droplets in the lower layer at the height of 11.09 m. At v = 2.5 m/s, the deposition reached 0.09 μl/cm^2^ only at the height of 8.84 m. Above 10.31 m, the deposition decreases rapidly to a minimum of 0.01 μl/cm^2^. By Tukey’s test, treatment 7 showed a highly significant difference between treatments 9 and 11 (*p*< 0.01).

At LAI = 1.65, the mean deposition was 0.04 and 0.06 μl/cm^2^ in only treatment 2 and treatment 4, respectively, and the rest of the treatments were similar and lower than 0.01–0.02 μl/cm^2^. By Tukey’s test, there was a highly significant difference (*p*< 0.01) in the mean deposition at v = 1.5 m/s and no significant difference (*p* > 0.05) among the treatments at v = 2.5 m/s. The operational parameters can be referred to according to the leaf area index when the focus of pests and disease control is on the trunk.

The mean CV value of deposition in each layer was used to express the penetration of droplet deposition, and the results showed that the CV value varied from 0.09 to 0.63. The higher the operating height, the worse the penetration at both speeds. For the same operating parameters, LAI = 1.65 was less penetrating than LAI = 1.01. The worst performance was at 11.09 m with a maximum of 0.63. At v = 1.5 m/s, the CV values were lower than 0.18 (LAI = 1.01) and 0.45 (LAI = 1.65) for heights below 10.31 m. in the analysis of the total deposition of the three layers, at LAI = 1.01, the flight altitude should be below 10.31 m (v = 1.5 m/s) and below 8.84 m (v = 2.5 m/s). For LAI = 1.65, v = 1.5 m/s and height below 10.31 m were recommended; the deposition effect was poorer when the height or the speed increased. From the overall view of canopy deposition, both LAIs of *A. catechu* at v = 1.5 m/s and below h = 10.31 m achieved relatively satisfactory deposition results, and this operation parameter was recommended.

### 3.2 Ground loss

**Figure 7:**
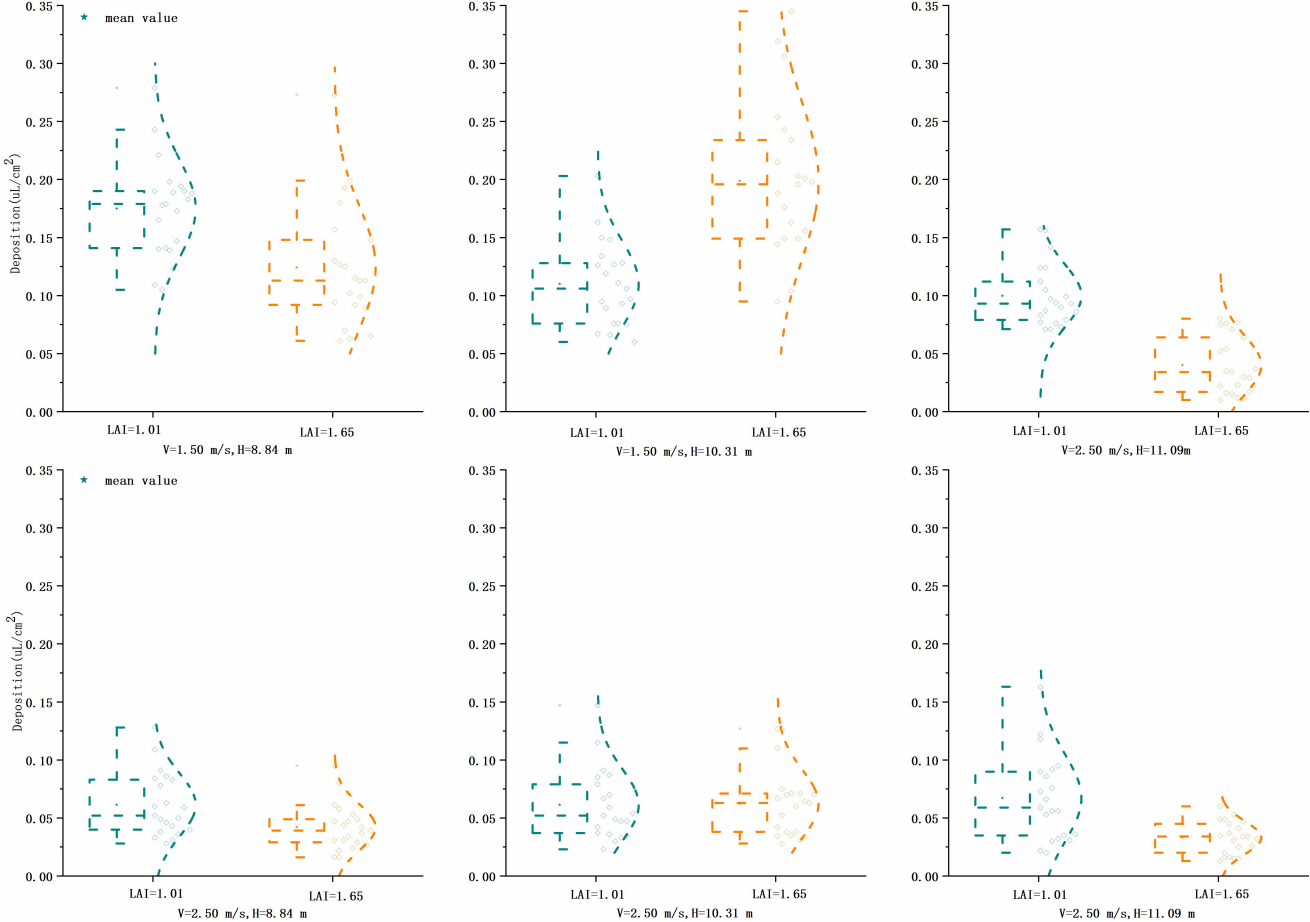
Comparison of the deposition of droplets at two flight heights and three flight speeds at three heights in the ground loss of two LAI plants. LAI, leaf area index.

**Figure 7** shows the sampling data of the ground loss area. By Tukey’s test, at v = 1.5 m/s, there was an extremely significant difference (*p*< 0.01) between ground loss deposition for LAI = 1.01 and LAI = 1.65 treatments at the same operating height. At v = 2.5 m/s, there was no significant difference (*p* > 0.05) between ground loss at the same operating height. At LAI = 1.01 and v = 1.5 m/s, there was an extremely significant difference (*p*< 0.01) in deposition between height 8.84 m and the other two heights; at v = 2.5 m/s, there was no significant difference (*p* > 0.05) between treatments in the deposition. At LAI = 1.65 and v = 1.5 m/s, there was a significant difference among all three heights (*p*< 0.05), and at v = 2.5 m/s, there was no significant difference among treatments (*p* > 0.05).

The mean value of deposition of each treatment was analyzed; at LAI = 1.01 and v = 1.5 m/s, the maximum mean value was 0.18 μl/cm^2^ at 8.84 m, and the other two heights were approximately 0.10 μl/cm^2^. At v = 2.5 m/s, the mean deposition value at three heights was approximately 0.06 μl/cm^2^. The difference was more apparent when LAI = 1.65 and v = 1.5 m/s, and the mean deposition was more significant at heights of 8.84 and 10.31 m; at up to 0.20 μl/cm^2^, the mean deposition decreased to 0.04 μl/cm^2^ when the height and velocity increased. Overall, the two LAIs of *A. catechu* had the most significant ground loss at v = 1.5 m/s, at heights 8.84 and 10.31 m. While the canopy deposition was also the largest and the penetrability was the best, it indicates that more droplets were deposited in the crop canopy and penetrated to reach the ground under this mode of operation. Due to the high flight altitude and speed, the rest of the treatment increased the droplet residence time in the air. The droplet trajectory was more susceptible to change by the external environment, and the deposition area was shifted. At the same time, plants with bigger LAI, thicker canopy, and more extensive total leaf area also led to less deposition, penetration, and ground loss. The field test has many uncontrollable factors, such as the crop growth pattern, flight attitude, and sampling point location. All the factors can improve the test results’ reference ability by increasing the sample size and providing referenceable data for spraying tall trees by UAV.

The coefficient of variation of deposition for each treatment of ground loss was relatively small, ranging from 7.64% to 52.90%, with an average of 27.04%. The coefficient of variation of canopy deposition was relatively large, a typical characteristic of field experiments. The middle canopy was the highest for CV value, with a mean of 69.53% and a maximum of 98.90%. The upper canopy had the second-highest coefficient of variation, with a mean of 46.83%. The lower canopy had the lowest coefficient of variation at 37.27%. It could assume that the sampling points in the middle of the canopy were affected by the upper canopy branches and pore distribution, and the unevenness of deposition distribution increased. The lower canopy was approximately 2 m away from the top of the canopy, and the sampling area reduced sharply. Hence, the droplet deposition could hardly reach this small area, and the droplet size was more selective, so the variation coefficient was insignificant. The sampling location of ground loss deposition includes the gap between plants and the bottom of plants; the gap allows the droplets to not be captured by the canopy and settle directly to the ground, with higher deposition and a smaller coefficient of variation.

**Figure 8:**
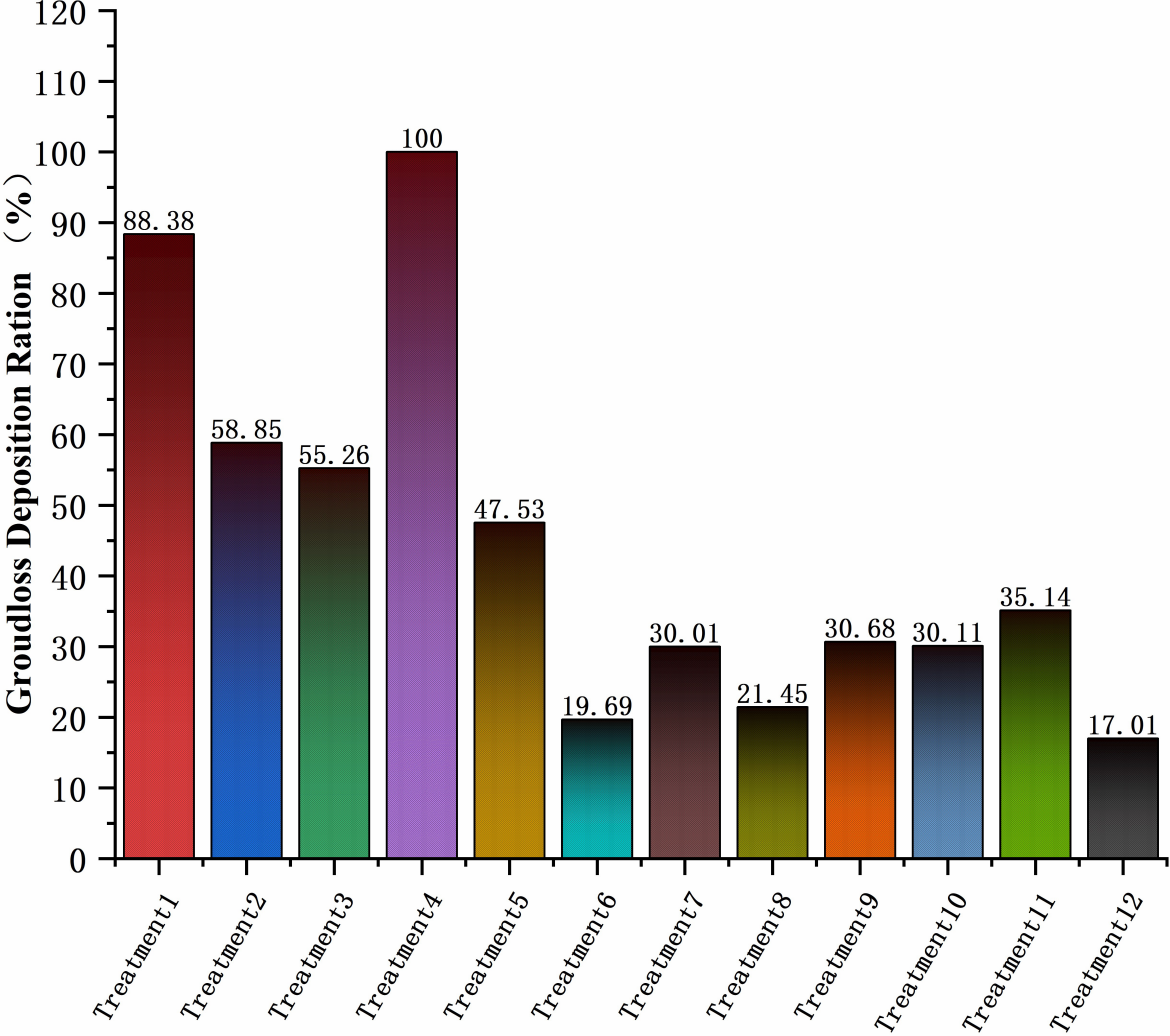
Percentage of total ground loss.

The deposition and location of sampling points for each treatment were plotted as a line graph and integrated to evaluate the total amount of ground loss deposition. Treatment 4 had the highest loss and set it as 100. For the other treatments compared with treatment 4, **Figure 8** shows the results that treatment 12 was the smallest, with only 17.01% of treatment 4. At v = 1.5 m/s, except for treatment 4, ground loss decreases with increasing operating height, and at v = 2.5 m/s, the ground loss was more similar across treatments. The total ground loss at v = 1.5 m/s was more than double that at v = 2.5 m/s. The lowest ground loss at a flight altitude of 11.09 m was presumed to be due to the high flight altitude, which caused some droplets to drift in the air. It was also indicated by the reduction in both canopy and ground deposition.

Comparing the deposition data between canopy and ground loss showed that both ground loss and canopy droplet deposition were lower at higher flight speeds and operating altitudes, which occurred on both plants with two different leaf area indices. When the UAV is rushing at a high altitude, the rotor wind field, especially the vertical downward airflow, makes the droplet movement and allows the time to become shorter. The droplet moving distance and time in the air increase, and the direction of movement is more likely to change, the ability of droplet deposition to the target becomes poor, and the application quality decreases.

### 3.3 Spray drift

**Figure 9:**
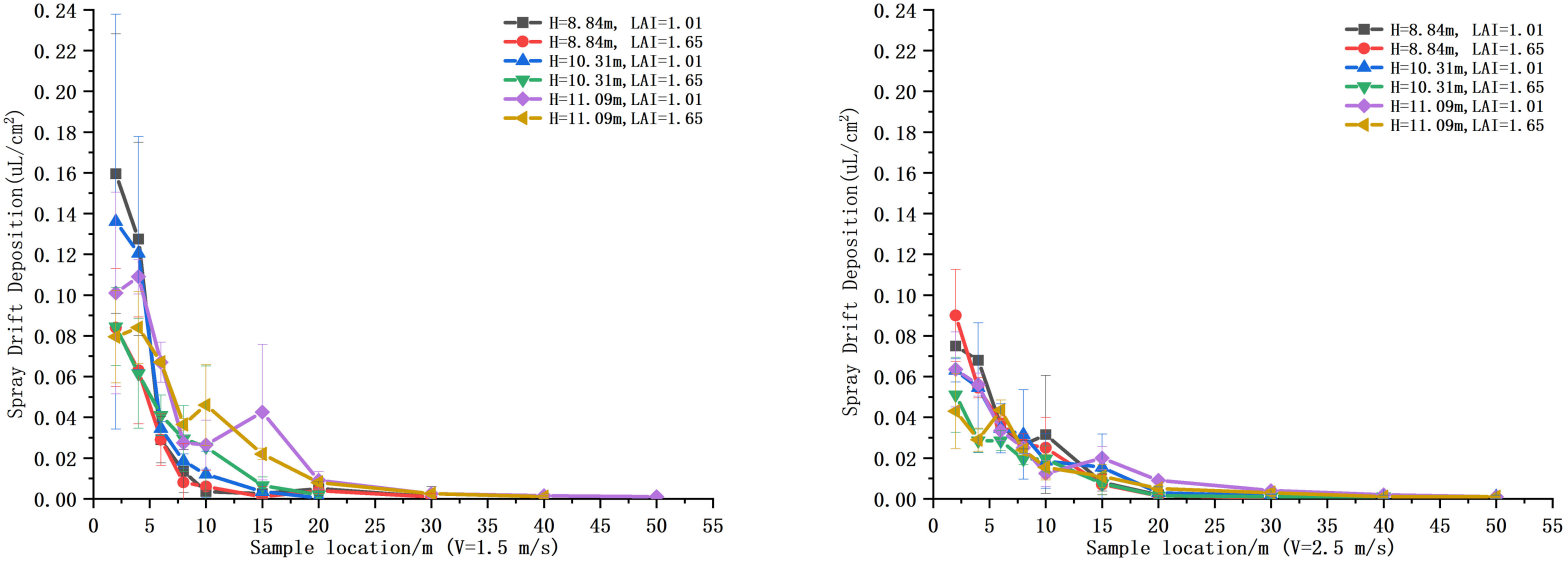
Deposition at each sampling site in the drift zone.

**Figure 10:**
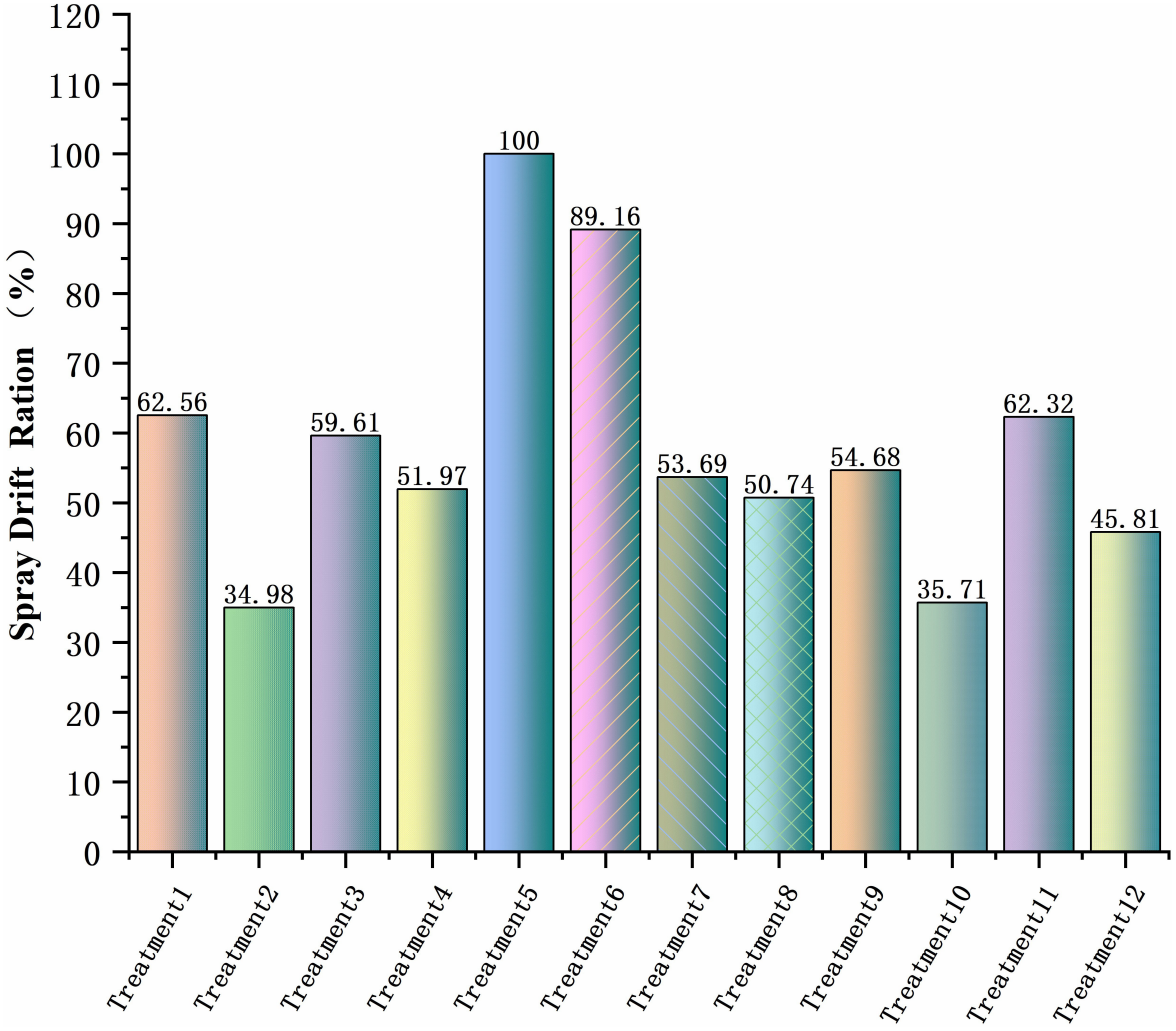
Percentage of spray drift.

**Figure 11:**
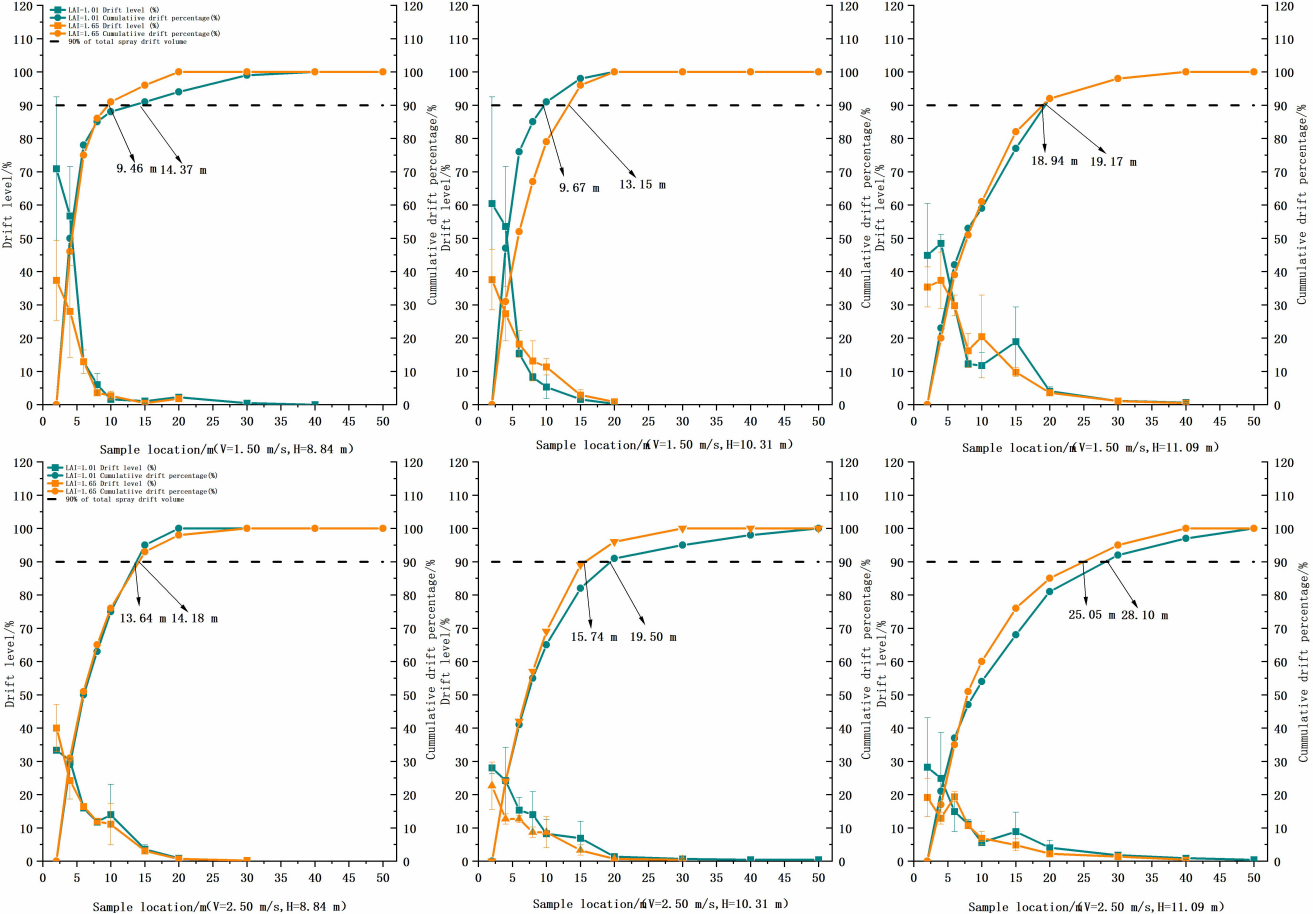
Comparison of the characteristic of downwind drift characteristic for each treatment at two flight heights and three flight speeds at three heights of two LAI plants. LAI, leaf area index.

The deposition of droplets in the drift sampling zone of each treatment was analyzed, as shown in **Figure 9**. There was no significant difference between the deposition of the 12 treatments by Tukey’s test (*p* > 0.05). The deposition was higher at 2 and 4 m in the drift zone, with a maximum of 0.16 μl/cm^2^, and decreased sharply after 10 m, below 0.04 μl/cm^2^. The percentage of deposition and spraying volume was calculated for each sampling point, and the total integral value of the deposition curve was used to evaluate the total drift of the test. The results showed that treatment 5 had the largest total drift, set at 100, and treatment 2 had the smallest, at 34.98. The ratio of each treatment to treatment 5 is shown in **Figure 10**. **Figure 11** shows the cumulative 90% total drift position. The locations of 90% of the total drift for treatments 1–12 were 9.46, 14.37, 9.67, 13.15, 18.94, 19.17, 13.64, 14.18, 15.74, 19.50, 25.05, and 28.10 m, respectively.

Comparing v = 2.5 m/s with v = 1.5 m/s, under the same operating parameters, the distance of 90% of the total drift increases, and the difference in the total drift was not too noticeable when the operating height was low. When the flight altitude increases to 11.09 m, the drift distance increases significantly; the maximum can be doubled. The total drift and 90% drift distance increase, so this flight altitude should be avoided. In addition, the ambient wind speed of this test was lower than 1.87 m/s, and the droplet drift was relatively small. According to the pineapple test by Wang et al. (2018), when the instantaneous wind speed increases to 4.7 m/s, the flight altitude is 3.5 m, and the farthest drift distance can reach 47 m. The drift problem caused by the ambient wind speed should be noticed for the application of UAV plant protection of tall trees.

## 4 Discussion

The initial kinetic energy of the droplets released at high altitudes gradually decreased with operating time, and the deposition area became bigger at lower altitudes. The droplet deposition uniformity, spray range, and penetration are mutually limited during UAV operation. The discussion of UAV spraying capabilities or configurations is irrelevant if canopy characteristics are not considered. Crop deposition largely depends on the crop growth stage; from the crop, the areca plant growth pattern was complex, and planting pattern, density, canopy depth, and pore structure would impact droplet deposition. There are relatively few reports on the application of UAVs on tall tropical crops. As the flight altitude increases, the canopy reflection effect decreases, the droplet flow velocity becomes more concentrated, and the turbulence is more stable than at a low altitude. According to the Tang et al. computational fluid dynamics simulation analysis of single-rotor UAVs (Tang et al., 2020; Tang et al., 2021), the higher the flight altitude, the larger the area of flow expansion along the lateral direction in the deposition area. For centrifugal nozzles, the spray quality depends mainly on the mechanical energy of the rotating atomizer. The electric motor runs at a certain speed, and the droplet size changes accordingly, and the multi-rotor UAV downwash flow field is more complex than that of the single rotor. The co-axial four-rotor wingtip vortices form a coupling effect under their respective rotors and in the crossover region, increasing the potential drift distance, especially at the flight altitude of 11.09 m. In contrast, the uniformity and penetration decreased, consistent with the findings of the six-rotor plant protection UAV studied by Zheng et al. (2018). The UAV operation was located approximately 10 m above the ground, obviously different from rice and wheat at approximately 3 m. The concentrated droplet group moves longer in the air. Due to the different factors, such as particle size, gravity, nozzle speed, ambient wind speed, temperature, and humidity, the droplet trajectory impacts the uncertainty. After the droplets reach the *A. catechu* canopy, leaf growth and development patterns, planting density, canopy height, density, and pore structure affect the trajectory of droplets.

In practical application, the UAV operating parameters should ensure the uniformity and penetration of droplet deposition. Wen et al. performed a study on a single rotor and showed that the simultaneous increase of speed and height also has an apparent effect on droplet drift (Wen et al., 2018). As the flight speed increases, the height of the spiral tail vortex formed by the wingtip vortex behind the fuselage becomes higher, and the higher the flight height, the longer the tail vortex lasts in the air. From the aircraft, it can be speculated that after the droplets were detached from the nozzle when the flight altitude was low, the deposition tended to be in a striped area. As the altitude increased, the striped area began to expand and was accompanied by a decrease in uniformity. When the flight altitude was too high, the droplets in the air stayed longer, subject to air traction and environmental and meteorological effects of the more significant disorder. Droplets may drift out of the target area, which needs further evaluation of spray volume distribution. Droplet deposition in the canopy and on the ground was relatively low at flight altitudes higher than 11 m. The UAV flight speed affects the droplet deposition to the canopy; it changes the droplet’s spray rate, air energy, and residence time; the slower the speed, the greater the air energy and the greater the penetration. The suitable speed can increase the residence time of droplets and concentrate air energy. The droplets are more easily deposited inside the canopy, but a too-slow speed may lead to branch closure, wasted spray, and compromised coverage.

The UAV model, nozzle type, planting method, environmental meteorology, and leaf formation of half and entire leaves affect droplet deposition and penetration. The ultimate goal of pesticide spraying is to provide sufficient coverage, reduce application volume, and control spray drift. Experiments have shown considerable variability in deposition and coverage under the same operational parameters for two different LAIs of *A. catechu*, so a study of the effect of areca growth morphology on droplet movement is the main direction of the following work. Given the high altitude of *A. catechu* and that adult *A. catechu* can reach 10 m or even tens of meters, the effect of operating height on deposition needs further study. As the crop density and growth develop during the later stage, the crop LAI will gradually increase, and the light transmission rate of each layer will decrease, increasing the difficulty of droplet deposition penetration to the middle and lower layers. The total droplet deposition in the canopy layer will decrease layer by layer, and the crop canopy layer will have a more noticeable effect on droplet interception. The sampling data of spray in the canopy, ground, and drift area can comprehensively reflect the distribution of spray quality, but there were also some limitations. The canopy sampling area was limited and did not accurately reflect the deposition of droplets within the canopy. The number of *A. catechu* sampled in this paper was 10, and the LAI of the selected *A. catechu* was close to that of the sampled trees. In practical application, the number of sampling cards should be increased appropriately according to the actual growth size of *A. catechu* to make the data more objective and accurate. The ground loss area sampling was more reliable in reflecting the droplet loss, and the drift area sampling can only reflect the far ground spray drift distribution. The measurement of drift in the air needs further improvement due to the test terrain’s limitation. Research on pesticide composition is also imminent (Hewitt et al., 2002). We also should conduct a relevant study on pesticide additives to change the droplet particle size by adding adjuvants to increase the deposition uniformity and reduce the drift potential. The application of UAVs in tall trees needs further research. In addition, modeling specific types of plant protection UAVs and tall trees to simulate droplet deposition distribution can further provide theoretical support for applying aerial spraying in tall trees.

The amount of aerial spray drift tends to decrease as the crop thrives, related to the canopy’s ability to capture droplets. The timing of airborne droplets varies with aircraft flight speed and ambient crosswind. Droplets from sidewind and headwind runoff may still be in the areca orchard, deposited into undetected areas, or on the ground evaporated or blown out of the orchard as aerial drift. Because airborne droplets are challenging to collect and quantify (Jensen and Olesen, 2014), sampling results can partially indicate the spraying effect. Studies on the movement trajectory of droplets during UAV operations need to be further enhanced. With plant heights of nearly 5 m in the *A. catechu* orchard, the wind speed in the orchard was slowed by the dense canopy. The smaller the droplet size, the more likely it was to be trapped inside the canopy. In contrast, finer droplets may be suspended in the air, aggregating into larger droplets or producing drift, and larger droplets are more likely to be deposited on the ground.

This research was based on an exploratory application of a specific quadrotor UAV model to *A. catechu* in a specific growth period. In this test, we used coated paper to visualize the effect of application, aided by ground and drift, to describe the effect of spray in many ways. These data support the potential application of UAV application technology to tall trees and provide recommendations for UAV application methods. The spray volume was 22.5 L/ha, which can be adjusted or repeated according to the actual application. The area of areca application during the fruit growth period may be concentrated in the trunk layer, which requires droplet size and deposition penetration. The UAV model, wingtip vortex, wake flow, ambient meteorology, nozzle type, boom position and length, droplet size, and operating parameters all pose challenges to the effective operation of the application. The current application of UAV models and droplet spray quality on tall trees must be supplemented by more field trial data. As designed for future planting systems, *A. catechu* planting structures should integrate spraying strategies.

## 5 Conclusion

This study describes a new application method to assess the credibility of a specific UAV type to apply to areca trees with different LAIs under different operational parameters. According to the analysis of the results of each treatment data, the flight height had a more significant effect on droplet deposition than speed and LAI, and the higher the height, the worse the overall penetration of droplets. The altitude had a significant effect on droplet penetration, with the CV values at up to 0.63. Comparing v = 1.5 m/s to v = 2.5 m/s, the coverage can be increased by more than 70%. The droplet deposition size and distribution uniformity in the bottom layer have significant differences, with UAV below 10.31-m height and 1.5 m/s speed, and the deposition penetration CV value could be controlled below 0.18 (LAI = 1.01) and 0.45 (LAI = 1.65). Ground loss at velocity 1.5 m/s was about twice as high as at velocity 2.5 m/s. When the operating height was 11.09 m, the ambient wind speed was 1.87 m/s, and the 90% drift distance increased to 28.10 m. The most critical issue is that this study provides more comprehensive data on the application of multi-rotor UAVs in tall trees. More plant protection-focused data are needed to confirm the feasibility of this new method of UAV application, and the deposition patterns of different droplet sizes within the *A. catechu* canopy need to be further explored.

## Data availability statement

The raw data supporting the conclusions of this article will be made available by the authors, without undue reservation.

## Author contributions

JW: conceptualization, methodology, software, formal analysis, investigation, and writing—review and editing. CM: investigation, writing—review and editing, and data curation. WY: conceptualization, investigation, and supervision. YY: visualization and investigation. TZ: formal analysis and data curation. SC: methodology and investigation. YL: supervision and project administration. PC: conceptualization, methodology, investigation, and writing—review and editing. All authors contributed to the article and approved the submitted version.
